# Sexually divergent DNA methylation patterns with hippocampal aging

**DOI:** 10.1111/acel.12681

**Published:** 2017-09-25

**Authors:** Dustin R. Masser, Niran Hadad, Hunter L. Porter, Colleen A. Mangold, Archana Unnikrishnan, Matthew M. Ford, Cory B. Giles, Constantin Georgescu, Mikhail G. Dozmorov, Jonathan D. Wren, Arlan Richardson, David R. Stanford, Willard M. Freeman

**Affiliations:** ^1^ Reynolds Oklahoma Center on Aging Oklahoma OK USA; ^2^ Department of Physiology University of Oklahoma Health Sciences Center Oklahoma OK USA; ^3^ Oklahoma Nathan Shock Center for Aging Oklahoma OK USA; ^4^ Oklahoma Center for Neuroscience University of Oklahoma Health Sciences Center Oklahoma OK USA; ^5^ Department of Biochemistry and Molecular Biology Pennsylvania State University University Park PA USA; ^6^ Department of Geriatric Medicine University of Oklahoma Health Sciences Center Oklahoma OK USA; ^7^ Division of Neuroscience Oregon National Primate Research Center Beaverton OR USA; ^8^ Arthritis & Clinical Immunology Program Oklahoma Medical Research Foundation Oklahoma OK USA; ^9^ Department of Biostatistics Virginia Commonwealth University School of Medicine Richmond VA USA; ^10^ Department of Biochemistry and Molecular Biology University of Oklahoma Health Sciences Center Oklahoma OK USA; ^11^ Oklahoma City VA Medical Center Oklahoma OK USA

**Keywords:** aging, divergence, DNA methylation, epigenetics, hippocampus, sex differences

## Abstract

DNA methylation is a central regulator of genome function, and altered methylation patterns are indicative of biological aging and mortality. Age‐related cellular, biochemical, and molecular changes in the hippocampus lead to cognitive impairments and greater vulnerability to neurodegenerative disease that varies between the sexes. The role of hippocampal epigenomic changes with aging in these processes is unknown as no genome‐wide analyses of age‐related methylation changes have considered the factor of sex in a controlled animal model. High‐depth, genome‐wide bisulfite sequencing of young (3 month) and old (24 month) male and female mouse hippocampus revealed that while total genomic methylation amounts did not change with aging, specific sites in CG and non‐CG (CH) contexts demonstrated age‐related increases or decreases in methylation that were predominantly sexually divergent. Differential methylation with age for both CG and CH sites was enriched in intergenic and intronic regions and under‐represented in promoters, CG islands, and specific enhancer regions in both sexes, suggesting that certain genomic elements are especially labile with aging, even if the exact genomic loci altered are predominantly sex‐specific. Lifelong sex differences in autosomal methylation at CG and CH sites were also observed. The lack of genome‐wide hypomethylation, sexually divergent aging response, and autosomal sex differences at CG sites was confirmed in human data. These data reveal sex as a previously unappreciated central factor of hippocampal epigenomic changes with aging. In total, these data demonstrate an intricate regulation of DNA methylation with aging by sex, cytosine context, genomic location, and methylation level.

## Background

Direct DNA base modifications, such as cytosine methylation (mC), are proposed to fundamentally regulate the mammalian genome through altering genome accessibility (Law & Jacobsen, 2010). Maladaptive changes in these epigenetic marks are potential drivers of pathogenesis and progression of many diseases (Robertson, 2005). In the central nervous system (CNS), epigenetic changes have been associated with a number of age‐related diseases, including Alzheimer's and cognitive impairment (Penner *et al*., 2010). Additionally, DNA modifications may regulate the gene expression program required for normal hippocampal learning and memory (Lister & Mukamel, 2015). Despite the focus on age‐related changes in mCG as a ‘biological clock’ (Horvath, 2013) and the potential importance of DNA modifications in CNS aging (Lardenoije *et al*., 2015), DNA methylation changes in CG and CH (i.e., non‐CG) contexts and genomic patterns with aging in the CNS of controlled animal models are largely unexplored. Additionally, studies have not examined the commonalities and differences between the sexes. The hippocampus is a central neural substrate of age‐related dysfunction and disease, but previous aging studies have not quantitatively examined mC genome‐wide with single‐base resolution. Recent findings, albeit in other tissues, provide support for the need to examine alterations in DNA modification with aging (Hahn *et al*., 2017; Petkovich *et al*., 2017; Stubbs *et al*., 2017; Wang *et al*., 2017).

In an effort to elucidate the functional role of DNA methylation alterations with aging in the hippocampus, we recently reported that neither the expression of the major DNA methylation regulating enzymes (DNA methyltransferases and ten‐eleven translocases) nor the total mean genomic methylation or hydroxymethylation levels in either CG or CH contexts change with aging in the male or female hippocampus (Hadad *et al*., 2016). These results were unanticipated given the number of recent reviews that emphasize global DNA hypomethylation with age across somatic and CNS tissues as a driver of the aging process (Chow & Herrup, 2015; Zampieri *et al*., 2015; Sen *et al*., 2016). A growing number of reports convincingly demonstrate that in the CNS, mC can increase or decrease at specific CG (Penner *et al*., 2016; Ianov *et al*., 2017) and non‐CG/CH (Mangold *et al*., 2017) sites and regions with aging. This suggests mechanisms whereby differential DNA methylation with aging is targeted in a locus‐specific manner. Thus, quantitative analyses with single‐base resolution are needed to identify where in the genome increases and decreases in methylation of cytosines are occurring with aging in the hippocampus and critically how sex factors into the epigenome response to aging.

This study sought to answer three questions: (i) Does global/total mean hippocampal methylation change with age; (ii) what are the comparative responses of hippocampal methylation patterns with age in males and females; and (iii) are there hippocampal lifelong sex differences in autosomal methylation? Using published recommendations on terminology (McCarthy *et al*., 2012), we define a sex difference as a difference between males and females that persists throughout the lifespan, and define sex divergence as a sex‐specific response to a stimulus, such as aging (Fig. S1). DNA methylation was quantified in a base‐specific manner across promoters, CG Islands and associated flanking regions, and gene regulatory regions from the hippocampus of male and female young (3 months) and old (24 months) C57BL6 mice using bisulfite oligonucleotide‐capture sequencing (BOCS), a method we have quantitatively validated (Masser *et al*., 2016). Targeting 109 Mb of the mouse genome (Hing *et al*., 2015) (over 30 million combined CG [~3 Million] and CH [~28 Million] sites) enables sequencing at depths exceeding coverage recommendations (Ziller *et al*., 2015) in multiple independent biological samples per group.

## Results

### Does global/total DNA methylation change with age?

DNA methylation was quantified in a base‐specific manner (i.e., methylation at each CG and CH was individually quantified) across almost all annotated promoters and CG Island units (Island, shore [± 2 kb from island] and shelf [± 2 kb from shores]) for a total of 109 Mb of coverage using bisulfite oligonucleotide‐capture sequencing (BOCS, Fig. S2A‐B). As the term CG island is often used nonspecifically to refer to just the CG island or the combination of CG island and the accompanying shores and shelves, we will use CG island (CGI) to refer to the island alone, and a CGI unit will be used to refer to the island, shores, and shelves together. BOCS was developed to focus sequencing to regions of interest and avoiding repeat sequences, thereby increasing sequencing depth, while avoiding the bias toward CG dense regions observed and fundamental to reduced‐representation bisulfite sequencing (Li *et al*., 2015; Masser *et al*., 2016). Over 30 million aligned reads were generated for each sample with a target enrichment of > 25‐fold from the whole genome for an average target sequence coverage of 20‐40X (Fig. S3). Using data from across all CG sites meeting sequencing coverage criteria, sample–sample correlations were consistently very high (*r* > 0.95), demonstrating robust reproducibility across all samples (Fig. S4).

Mean levels of DNA methylation of all the CG and CH site calls in the targeted portion of the genome were calculated, and there was no observed difference in global/total mean methylation levels across the hippocampal genome with age in males or females in either CG or CH contexts (Fig. S5A and D). Furthermore, a consistent bimodal distribution of CG methylation was observed in both sexes at both ages (Fig. S5B). No differences in the distribution of CG methylation in promoter and CGI units were evident as well (Fig. S5). Methylation at CH dinucleotides in the CNS has previously been shown to be higher relative to peripheral tissues (Lister *et al*., 2013; Lister & Mukamel, 2015; Mangold *et al*., 2017), and similarly, we observe large numbers (> 250 000) of individual CH sites (~1% of all CH sites quantified) with high methylation levels (> 10%) in the hippocampus (Fig. S5E). While we have previously quantified the whole‐genome levels of CH methylation in the hippocampus (Hadad *et al*., 2016), our previous approach did not yield base‐specific data. This was also evident in the density distributions of CH sites subdivided into promoter regions or CGI units (Fig. S5F). In total, there was no evidence for a genome‐wide loss in methylation in either CG or CH sites, including when subdivided into promoter and CGI units.

### What are the comparative responses of mC to aging in males and females?

**Figure 1:**
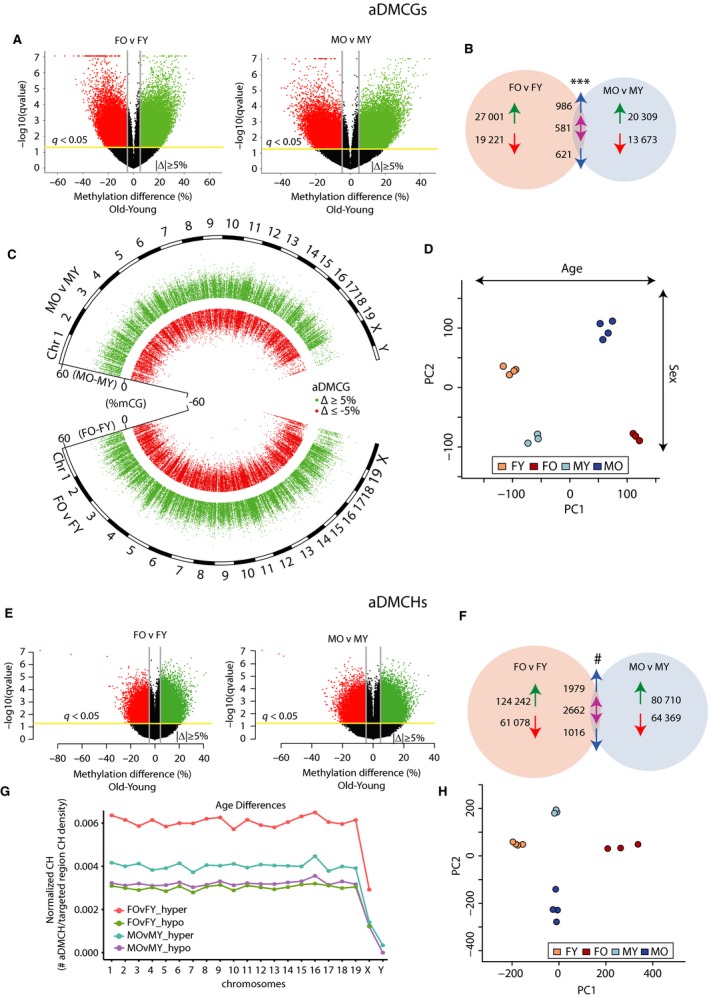
Age‐related differentially methylated CGs (aDMCG) and CHs (aDMCGH) in male and female hippocampus. A) Volcano plots of pairwise comparisons of age‐related CG sites methylation changes in females (Female Old – FO vs. Female Young FY) and males (Male Old – MO vs. Male Young MY). Sites with false discovery‐corrected p‐value (*q* < 0.05) and an absolute magnitude change Old‐Young > |5%| were called differentially methylated. Sites with an age‐related increased methylation are represented in green and those with decreased methylation in red. B) Comparing the aDMCGs in males and females, more sites were found in common between the sexes than would be expected by chance (***, *P* < 2.6E‐106), but the majority of aDMCGs were sex‐specific. Arrows represent increased or decreased methylation with age, and in the intersection, blue arrows represent common regulation between the sexes, and the double purple arrow represents different direction of change in the sexes with age. C) aDMCGs are presented by chromosomal location in males (top) and females (bottom) and the difference in mean methylation (Old‐Young) on the inner axis. Each point represents one aDMCG meeting false discovery rate (FDR) cutoff of *q* < 0.05. Sites ≥ 5% absolute change in methylation with age (hypermethylated) are in green, while in red are sites ≤ −5% change in methylation with age (hypomethylated). D) The union of sites from B were used in a principle component analysis of the samples. Samples cluster by group and separated by age in the 1st component and by sex in the 2nd component. E) aDMCHs were compared in the same manner for differences in methylation with age in females and males. F) More sex‐common aDMCHs were observed than expected by chance (#, *P* < 01E‐200), but the majority of aDMCHs were specific to one sex or the other. Arrows as in B. G) Distribution of CH sites across the chromosomes examined by comparing the number of aDMCHs per the number of CHs in the covered regions of that chromosome. Equal representation of aDMCHs across the autosomes was observed with a lower rate of aDMCHs in the sex chromosomes. H) Taking the union of the sites in F, principle component analysis of the samples demonstrated tight clusters of samples by sex and age.

Next, whether specific sites in the genome were differentially methylated with aging in the female and male hippocampus was investigated. Age‐related changes in mC were determined in a site‐specific manner in males and females at individual CG (aDMCGs) and CH (aDMCHs) sites. aDMCGs were equally distributed between hypermethylation and hypomethylation events (Fig. 1A, Data S1). Comparing the aDMCGs in males and females, the majority (> 90%) were sexually divergent (Fig. 1B and Data S1) although the extent of overlapping sites was greater than expected by chance (hypergeometric test, *P* < 2.6E‐106). This leaves the potential that different sites change in methylation level with aging in males and females, but that these sites could be located in close proximity. A nearest neighbor analysis was performed between aDMCGs in males and females, and the average distance was found to be > 4kB between sex‐specific aDMCGs in males and females. Differential methylation of CG sites with aging was observed across the genome (Fig. 1C). Taking the union of all the aDMCGs in males and females, samples were clustered and formed tight groupings based on age and sex (Fig. 1D), further demonstrating the consistency of the methylation patterns. Animals separated by age in the 1st component and by sex in the 2nd component.

We have previously observed CNS tissue differences in MHCI promoter CH methylation with aging (Mangold *et al*., 2017), but there is little additional reported data on CH methylation with aging in the CNS (Lister *et al*., 2013). Age‐related differentially methylated CHs (aDMCHs) were identified in a manner similar to aDMCGs. Hypermethylated and hypomethylated aDMCHs were observed with aging in males and females, with a greater number of hypermethylated sites (Fig. 1E). Comparing the aDMCHs in males to females, the majority were sex‐specific but with greater overlap than would be expected by chance (hypergeometric test, *P* < 01E‐200) (Fig. 1F). Unlike aDMCGs, aDMCHs in males and females are closer to one another as > 65% of these sites are within 1 kb of each other, likely due to the number of aDMCHs and the frequency of CHs in the genome compared to CGs. aDMCHs were found across the autosomes at a roughly equal density (Fig. 1G). Clustering of samples by aDMCHs demonstrated close grouping of samples and separation of the groups by age and sex (Fig. 1H).

**Figure 2:**
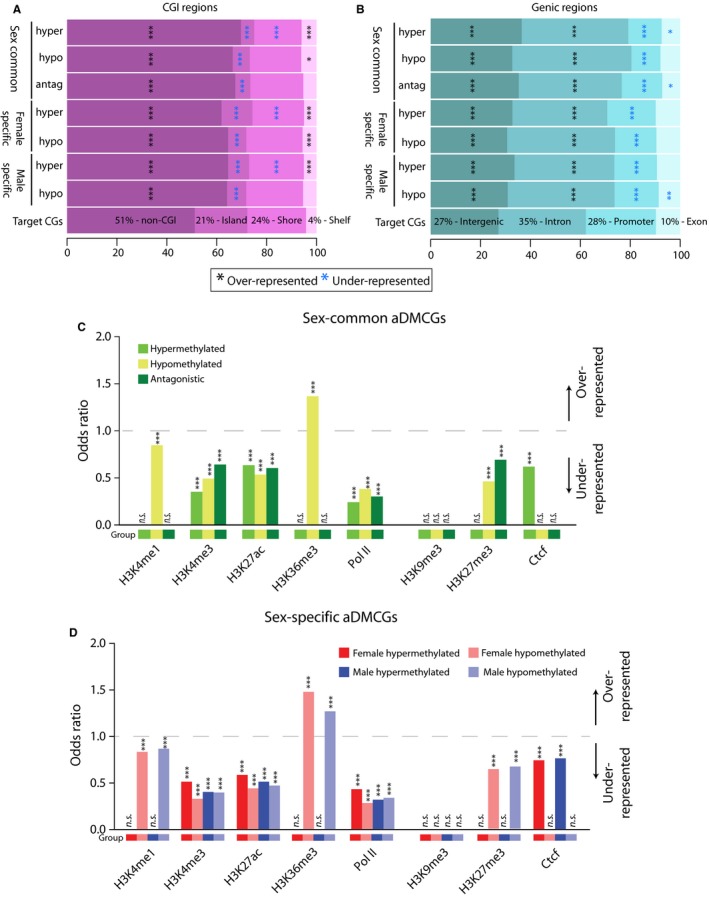
Annotation enrichment patterns of age‐related differentially methylated CG sites. A) Sex‐common and sex‐specific aDMCG distributions were examined for enrichment in relation to the CG distribution in the CGI unit regions analyzed (target). aDMCGs were separated by whether they decreased or increased in methylation with aging or if they were antagonistically differentially methylated sex‐common sites (hypermethylated with aging in one sex and hypomethylated in the other sex). Over‐representation of non‐CGI regions and under‐representation of Islands was observed for all comparisons. B) Similarly, genic regions were examined, and aDMCGs were found to be over‐represented in intergenic and intronic regions while under‐represented in promoter regions. (***P < 0.001, **P < .01, *P < 0.05 χ^2^ analysis, coloring by over‐representation, black or under‐representation, blue.) C) Odds ratios demonstrating enrichment of sex‐common aging differentially methylated CG sites (aDMCGs) for ENCODE and regulatory elements (activation – H3K4me1, H3K4me3, H3K27ac, H3K36me3, PolII and repression – H3K9me3, H3K27me3, Ctcf) by GenomeRunner analysis. Enrichment comparisons were carried out for hypermethylated aDMCGs (light green), hypomethylated aDMCGs (yellow), and antagonistic (dark green) sex‐common aDMCGs. Odds Ratios greater than 1.0 (gray dotted line) demonstrate over‐represented while those less than 1.0 are under‐represented. Significant enrichment or depletion is denoted by stars where *P < 0.05, **P < 0.01, and ***P < 0.001. D) Odds ratios for sex‐specific aDMCGs enrichment for ENCODE and regulatory elements. Enrichment comparisons were carried out in each sex for hypermethylated aDMCGs (dark red – females, dark blue – males) and hypomethylated aDMCGs (light red – females, light blue – males). Odds ratios greater than 1.0 (gray dotted line) are over‐represented while those less than 1.0 are under‐represented. Significant enrichment or depletion is denoted by stars where *P < 0.05, **P < 0.01, and ***P < 0.001.

In other organs, aging CG methylation changes demonstrate enrichment within common annotated genomic elements including CGIs, CGI shores, exons, and around transcriptional start sites (McClay *et al*., 2014; Bell *et al*., 2016). Sex‐common aDMCGs and sex‐specific aDMCGs in males and females were assessed for over‐representation in genic (promoter, intron, exon) and CGI unit (island, shore, shelf) locations as compared to the distribution of CGs targeted in the oligonucleotide capture set for which there was coverage meeting our cutoffs. Enrichment of aDMCGs in outside of CGI units, in intergenic regions and in introns, was evident (Fig. 2A,B). CGIs and promoter regions were under‐represented as were CGI shores, among hypermethylation events. Of note, while the specific regions or sites differentially methylated with aging are largely sex‐specific, they demonstrate similar patterns of genic and CGI unit over‐/under‐representation in sex‐common and sex‐specific age‐related changes regardless of whether the sites were hypomethylation or hypermethylation events. These results differ from those found in non‐CNS tissue (McClay *et al*., 2014; Bell *et al*., 2016) and demonstrate non‐CG dense intergenic and intronic regions as preferential locations for age‐related hippocampal epigenetic alterations, while CG dense regions such as CGIs and promoters are under‐represented in age‐related changes in methylation. Additionally, there was not a significant difference in the localization of sex‐common and sex‐divergent differences with aging.

Enrichment of aDMCGs in enhancer regions was also performed against ENCODE datasets of mouse brain tissue enhancer locations using GenomeRunner (Dozmorov *et al*., 2012) (Fig. 2C,D). Regions associated with active transcription, H3K4me3, H3K27ac, and PolII, were generally under‐represented as a location for aDMCGs, regardless of the whether the site was hyper‐ or hypomethylated with aging. Sex‐common and sex‐specific hypomethylated sites were enriched at the exonically localized H3K36me3 and under‐represented at the primed enhancer marker H3K4me1. The repressive mark H2K27me3 was under‐represented in hypomethylation aDMCGs, while sex‐common hypomethylation events and sex‐specific hypermethylated sites were under‐represented in Ctcf regions. Sex‐specific aDMCGs demonstrated similar patterns of enhancer enrichment in males and females, though, with slight differences in the odds ratios (Fig. 2D).

**Figure 3:**
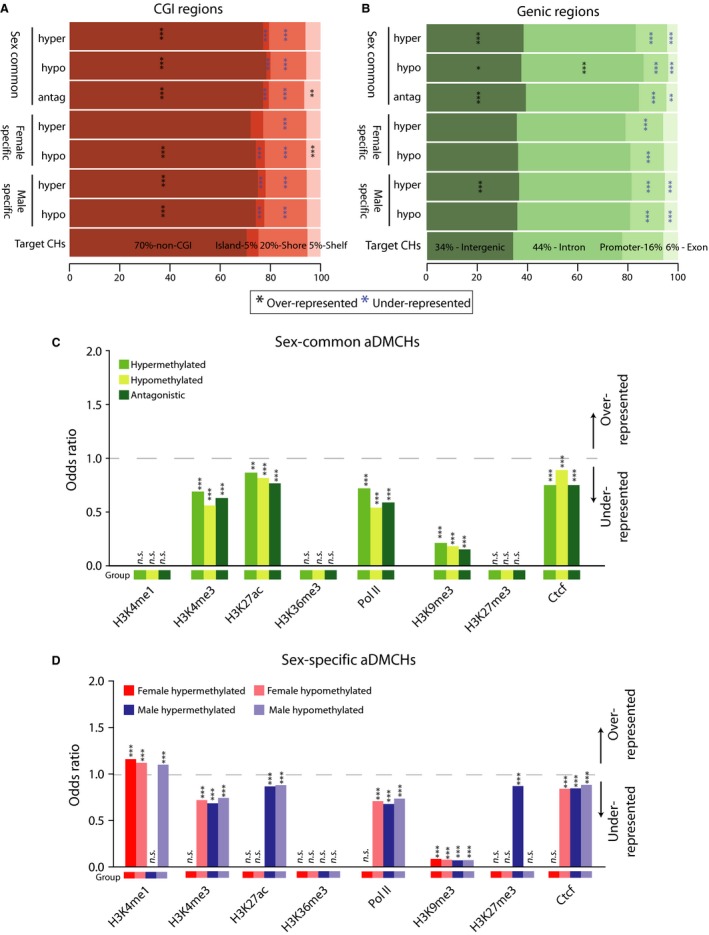
Annotation enrichment patterns of age‐related differentially methylated CH sites. A) aDMCH distributions for males and females in the CGI unit regions analyzed (target) demonstrated over‐representation in non‐CGI regions and under‐representation in CGI shores and islands themselves (with one exception). In many cases, CGI shelves were over‐represented for aDMCHs. B) In genic regions, aDMCHs were found to be over‐represented in intergenic and under‐represented in promoter and exonic regions. For a number of groups, especially hypomethylation, introns were over‐represented as well. (***P < 0.001, **P < .01, *P < 0.05 χ^2^ analysis, coloring by over‐representation, black or under‐representation, blue.) C) Odds ratios of sex‐common aDMCHs for ENCODE and regulatory elements by GenomeRunner analysis. Enrichment comparisons were carried out for hypermethylated aDMCHs (light green), hypomethylated aDMCHs (yellow), and antagonistic (dark green) sex‐common aDMCHs. Odds ratios greater than 1.0 (gray dotted line) are over‐represented while those less than 1.0 are under‐represented. Significant enrichment or depletion is denoted by stars where *P < 0.05, **P < 0.01, and ***P < 0.001. D) Odds ratios of sex‐specific aDMCHs enrichment for ENCODE and regulatory elements. Enrichment comparisons were carried out by sex for hypermethylated aDMCHs (dark red – females, dark blue – males) and hypomethylated aDMCHs (light red – females, light blue – males). Odds ratios greater than 1.0 (gray dotted line) are over‐represented while those less than 1.0 are under‐represented. Significant enrichment or depletion is denoted by stars where *P < 0.05, **P < 0.01, and ***P < 0.001.

aDMCHs demonstrated similar but not identical patterns of enrichment for genomic elements. aDMCHs were under‐represented in CGIs (with the exception of hypermethylation aDMCHs in females) and shores while enriched in non‐CGI unit regions (Fig. 3A). In genic regions, aDMCHs were under‐represented in promoter and exonic elements while enriched in intergenic and in most comparisons intronic regions (Fig. 3B). Much like sex‐common aDMCGs, sex‐common aDMCHs were significantly under‐represented among activation‐associated enhancer elements H3K4me3, H3K27ac, and PolII and the repressive marks H3K9me3 and Ctcf (Fig. 3C). However, unlike aDMCGs, sex‐specific aDMCHs were over‐represented at active H3K4me1 marks, and no enrichment was found at H3K36me3‐associated regions (Fig. 3D).

### Are there lifelong sex differences in autosomal methylation?

**Figure 4:**
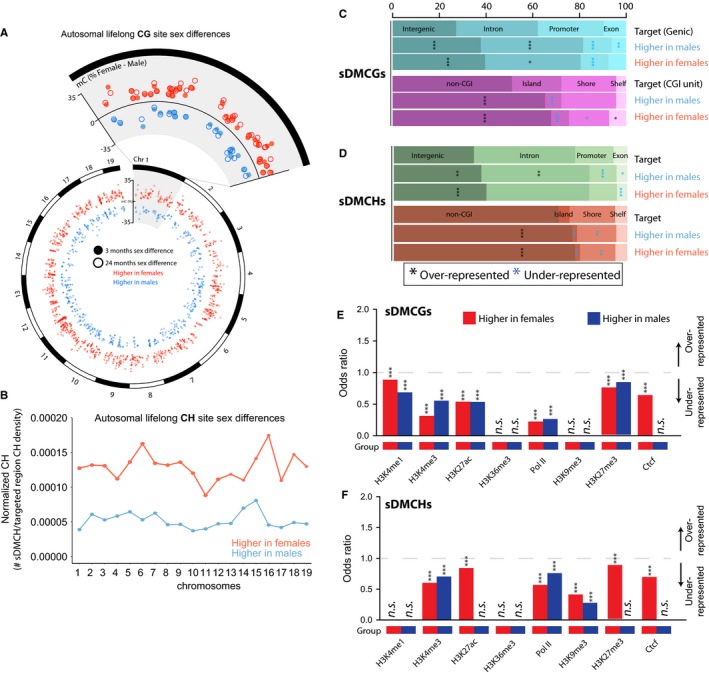
Lifelong sex differences in autosomal DNA methylation. A) Autosomal locations of the 901 lifelong CG site sex differences (sDMCGs) with higher methylation levels in females (orange) and sites that have higher methylation in males (blue). Closed circles represent the methylation difference between sexes in young (3 months) mice, while open circles represent the methylation difference between sexes in old (24 months) mice. For clarity, chromosome one is enlarged to visualize the lifelong nature of these autosomal sex differences. B) Autosomal distribution of lifelong CH site sex differences (sDMCHs) for the 3 028 CH sites that have either higher methylation levels in females (orange) or higher methylation in males (blue) relative to the CH density of the region examined. C) sDMCG and D) sDMCH site enrichment profiles among Genic (top) and CGI unit (bottom) regions for sites with higher methylation levels in males or in females. Percentages of sDMCGs and sDMCHs in each region type are presented in Figs. 2 and 3 (***P < 0.001, **P < .01, *P < 0.05 χ^2^ analysis, coloring by over‐representation, black or under‐representation, blue). E) Odds ratios of sDMCG and F) sDMCH enrichment in ENCODE and regulatory elements by GenomeRunner analysis. Odds ratios greater than 1.0 (gray dotted line) are over‐represented while those less than 1.0 are under‐represented. Significant enrichment or depletion is denoted by stars where * *P* < 0.05, **P < 0.01, and ***P < 0.001.

While sex differences in methylation patterns are evident during development (Nugent *et al*., 2015), little is known about whether brain sex differences persist throughout life. Sex differences in methylation of 901 CG (sDMCG) and 3 028 CH (sDMCH) sites were found at both young and old ages (e.g., higher in males than females at both young and old age, or vice versa). Analysis of sex differences was restricted to autosomes, and sex differences were found throughout the autosomes for both CGs (Fig. 4A) and CHs (Fig. 4B, Data S2). The difference in methylation between males and females was also found to be stable between young and old ages at these sites. These lifelong sex differences in CG and CH site methylation were significantly enriched in intergenic, intronic, and non‐CGI unit regions while generally under‐represented in promoters, exons, CGIs, and shores, with some exceptions (Fig. 4C and D). Additionally, CH sites that were higher in males were significantly enriched in introns, while the CH sites with higher methylation in females were not (Fig. 4D). For both sDMCGs (Fig. 4E) and sDMCHs (Fig. 4F), sex differences were not enriched for either active or repressive enhancer elements. These findings demonstrate that while not extensive, sex differences in hippocampal CG and CH methylation are evident in early adulthood (3M) and persist into advanced age.

### Replication in humans

**Figure 5:**
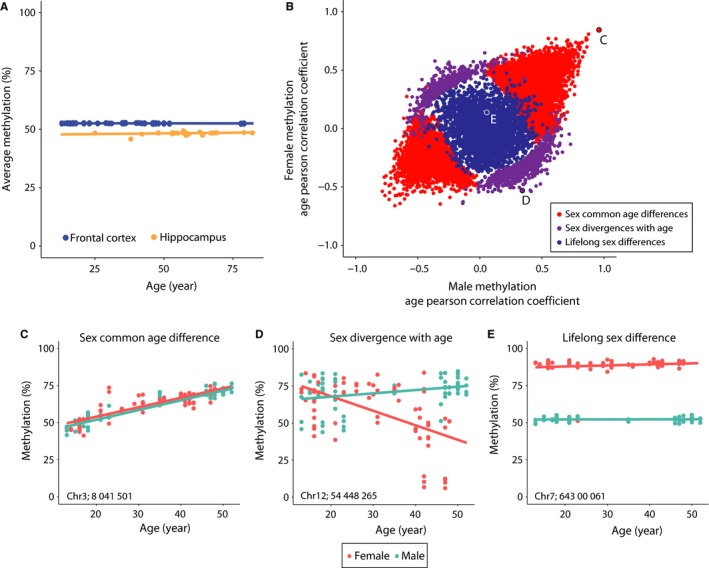
Methylation changes with aging and sex differences in the human central nervous system (CNS). A) Publicly available human methylation data from hippocampus and frontal cortex demonstrate no change in mean CG methylation with age. B) Using the fontal cortex data, for which there is a larger samples size and equal distribution between sexes, a general linear model was used to determine individual sites with significant age, sex, or interaction effects on methylation level. Plotted by Pearson's correlation coefficients to age by females (*y*‐axis) and males (*x*‐axis), sites with sex‐common age‐related decreases in methylation (red, bottom left) and increases in methylation (red, top left) are evident. Sites with sexually divergent response to aging (significant interaction effect) are in purple. Lifelong sex differences are plotted in blue. Sites without any significant factor of age or sex are not shown to improve clarity. C) Example site of a sex‐common age‐related differentially methylated site. D) Example of sexually divergent response to aging. E) Example of lifelong sex difference. Locations of specific sites are given and are also highlighted in panel B.

To validate the primary findings of the mouse studies, namely lack of hypomethylation with age, sex‐common and sex‐divergent differences with aging, and lifelong sex differences, we collected publicly available datasets from control human brain samples across a range of ages. Data from 19 hippocampal and 145 frontal cortex control (nondiseased) samples aged 13‐95 were collected. These data were generated with methylation microarrays and provide CG methylation quantitation across ~450 000 probes. When assaying hypomethylation with age, a linear model of age's effects on mean methylation of any sample (methylation ~ age) showed neither a significant effect in hippocampus (*P* = .228, *R* ^2 ^= .07185) nor in frontal cortex (*P* = .543, *R* ^2 ^= .002587) (Fig. 5A). As sufficient sample size in males and females was not available for hippocampus, frontal cortex data (69 females and 70 males) were used to determine specific differential methylation with aging. Sites were analyzed for the factors of sex and age and for interaction effects (methylation ~ age, methylation ~ sex, methylation ~ sex:age). A majority of sites (22 204) demonstrated a main effect of age alone, 2 723 were found to have a main effect of sex alone, and 4 154 sites showed both sex and age effects with a significant interaction. The sites with only an aging effect were attributed to sex‐common age‐related changes, while sites with only an effect of sex were sex differences. A significant interaction effect was indicative of sites with age‐related sex divergences. As presented in Fig. 5B, these effects were generally evenly distributed – increases and decreases in methylation with age and sites with higher methylation in males or in females. Examining examples of each phenotype, clear sex‐common age changes (Fig. 5C), sex divergences with aging (Fig. 5D), and autosomal (sites were limited to only autosomes) lifelong sex differences (Fig. 5E) were observed. These human data collected and analyzed by different methods than the mouse data presented here demonstrate that the general principles of the mouse findings also occur with aging in the human CNS.

## Discussion

This is the first comprehensive genome‐wide and base‐specific quantitation study of mC patterns and alterations with age in the male and female hippocampus. A delineation of the patterns of age‐related changes in DNA methylation is necessary for understanding both the potential functional effects of these changes and the regulation of DNA methylation patterns with aging. This study demonstrates a number of important principles for hippocampal DNA methylation: (i) genome‐wide hypomethylation with aging does not occur; (ii) site‐specific differential methylation of both CG and CH sites occurs with aging; (iii) males and females share some sites of differential methylation, but age‐related changes are primarily sexually divergent; (iv) lifelong autosomal sex differences in CG and CH methylation are evident; and (v) these findings are replicated in human CNS data.

### Genome‐wide hypomethylation with aging

No decreases in genome‐wide CG or CH methylation were evident with aging in this study in either mice or humans. It is often stated that DNA methylation decreases globally with age across tissues (Ashapkin *et al*., 2015; Chow & Herrup, 2015; Xu, 2015; Zampieri *et al*., 2015; Sen *et al*., 2016) on the basis of reports using older chromatographic methods with low replicate numbers and no statistical analyses (Wilson & Jones, 1983; Singhal *et al*., 1987). With modern sequencing tools, this long‐standing hypothesis of an age‐related loss in global methylation can be revisited with more quantitatively accurate and validated tools. Taken with our previous findings of no changes in mean hippocampal DNA methylation with aging as determined by low coverage whole‐genome oxidative bisulfite sequencing and pyrosequencing of LINE and SINE repeat elements (Hadad *et al*., 2016), genomic hypomethylation with aging is not evident in the hippocampus. Studies published since the submission of this report examined liver, lung, heart, and cortex using bisulfite sequencing methods have also found no genome‐wide hypomethylation with aging (Cole *et al*., 2017; Stubbs *et al*., 2017; Wang *et al*., 2017).

### CG and CH methylation are regulated with aging

A principle finding from this study is that CH methylation changes in a base‐specific fashion between adulthood and old age. Prior brain aging and methylation studies have not examined CH, also referred to as non‐CpG, methylation. Large numbers of CH sites were differentially methylated with aging, over 100 000 in both males and females. Widespread methylation (> 10% mC) of CH sites agrees with previous findings that the CNS contains some of the highest mCH levels in the body (Lister *et al*., 2013). CG dinucleotides are under‐represented in the mammalian genome, while CH contexts are much more common (~30 fold more) as evidenced by the targeted regions containing over 28 million cytosines in the CH context, and approximately 3 million in the CG context. The reproducible methylation of specific CH sites we observed here and that has been previously reported (Mangold *et al*., 2017) argues against accidental or nonregulated methylation of these sites. The functional role of CH methylation in the mammalian genome is only beginning to be understood and requires further study (He & Ecker, 2015). Given the important developmental role of mCH in synapse development, dysregulation of mCH with aging could have important functional impacts (Lister *et al*., 2013). Additionally, as mCH has differences in writing, erasing, and reading mechanisms as compared to mCG, altered methylation at CH sites could have functionally distinct impacts from CG methylation changes (Kinde *et al*., 2015; Mo *et al*., 2015). These data provide the first view, to our knowledge, that specific CH sites across the genome are differentially methylated with aging in the hippocampus. When possible, such as with bisulfite sequencing approaches, inclusion of mCH analysis in brain aging studies is warranted, and future cell type‐specific studies will be able to determine whether aDMCHs are preferentially occurring in neurons, as neurons have higher levels of mCH than non‐neuronal cell types in the brain (Lister *et al*., 2013).

### Sex divergence of differential methylation with aging

Prior studies of age‐related changes in brain cytosine methylation have generally not examined the factor of sex in the patterns of changes. While there are sex divergences in methylation during development (Nugent *et al*., 2015) and with aging in MHCI promoters (Mangold *et al*., 2017), the base‐specific differences in methylation changes with aging across the genome in both sexes have not been examined [Fig. S1 for graphical definitions of sex divergence and difference according to (McCarthy *et al*., 2012)]. While males and females share many more sex‐common age‐related changes than would be expected by chance, the majority of aDMCGs and aDMCHs were sexually divergent. Our findings reveal a previously unappreciated central factor of sex in age‐related epigenetic changes. Importantly, this sexual divergence was not simply the result of a loss in sex differences with aging, which were observed to persist throughout life as also evident in the human analysis. A recent report (Stubbs *et al*., 2017) found mouse methylation ‘ages’ are similar in males and females and that sex hormones influence methylation ‘age’. Taken together with our results, it is clear that the epigenomic response to aging is a combination of sites regulated in both sexes and sites regulated in only one sex. The enrichment of aDMCGs and aDMCHs in annotated genomic regions and enhancers is quite similar providing evidence that these sex divergences are not generally occurring in different types of genomic locations, rather there is some form of spatial regulation by sex on the exact location of aDMCGs and aDMCHs. Future studies of epigenomic changes with brain aging will need to incorporate both males and females into study designs, and sex‐specific databases of enhancer locations need to be developed to improve interpretation of these data.

### Enrichment of age‐related changes to annotated genomic regions

Recent mouse studies in liver and other tissues examining age‐related methylation changes find limited agreement in the types of genomic regions (*e.g.,* introns, exons, promoters) where differences are likely or unlikely to occur (Cole *et al*., 2017; Hahn *et al*., 2017; Stubbs *et al*., 2017), and aDMCGs observed here share commonalities and differences in enriched regions with these studies. Therefore, we focused on comparing the genomic enrichment between aDMCGs and aDMCHs, males and females, and between hypermethylated and hypomethylated sites. Enhancers have been identified previously to be genomic regions with dynamic DNA methylation with aging (Jones *et al*., 2015). For example, the presence of H3K27ac and H3K4me1 marks is indicative of active enhancer regions, while H3K4me3 marks are enriched in active promoters (Chen & Dent, 2014). The presence or absence of H3K27ac at enhancer regions distinguishes enhancers as active or inactive/poised, respectively (Creyghton *et al*., 2010). Interestingly, H3K36me3 is suggested to define exon boundaries and play a role in exon selection during transcription (Schwartz *et al*., 2009). Together the relative enrichment or depletion of these different genomic regions demonstrates that the brain has a unique pattern of regions where age‐related methylation changes are likely or unlikely to occur compared to other tissues. It should be noted that ENCODE datasets were generated against young male mice (Mouse Ecode Consortium *et al*., 2012). Age‐ and sex‐specific enhancer annotations as well additional data on chromatin structure will aid in placing methylation changes in their appropriate context (Pal & Tyler, 2016). This is emphasized by the finding that the genomic regions enriched in age‐related methylation changes differ from other tissues.

### Lifelong autosomal sex differences

While more limited in number than age‐related differences, lifelong autosomal CG and CH methylation is evident in the mouse hippocampus. Developmental sex divergences in CNS methylation have been recently reported (Nugent *et al*., 2015). Here, we observe an additional phenomenon – differences between males and females that are evident after development and are stable throughout life. While differences in sex chromosome methylation and *X*‐ and *Y*‐encoded transcript expression are well known, these data demonstrate a further level of autosomal sex differences. This was found to be true in both mice and humans. The functional implications of lifelong autosome sex differences remain to be determined, and sex differences in CH methylation have, to our knowledge, not been previously reported. sDMCGs and sDMCHs were enriched in intergenic, intronic, and non‐CGI unit regions while under‐represented in CG islands and most of the enhancer regions examined. As stated previously, integration of these data with enhancer elements remains difficult due to the lack of sex‐specific enhancer data for comparison and is an area of needed study.

### Future studies

These findings raise important questions to be addressed in future studies. (i) These findings will need to be replicated in independent mouse experiments and across mouse strains. (ii) The epigenomic regulatory processes that could lead to the profound sex divergence with aging observed are unknown. While epigenetic mechanisms establishing methylation patterns during organismal development have been extensively studied, the mechanisms whereby methylation or demethylation is directed to specific genomic locations with aging are not known and require investigation. Potential mechanisms include hormonal modulation of DNMT activity, which may explain sex differences with aging and sexual‐divergent epigenetic responses to aging, but the signal gating that targets changes to specific loci requires extensive further investigation. The sexual divergence with aging described here could potentially be harnessed to help understand these regulatory processes as these differences are naturally occurring and not the result of a genetic intervention. (iii) While this study did not distinguish between mC and hmC, future studies will need to identify how hydroxymethylation changes within and between sexes with aging using oxidative bisulfite sequencing (Hadad *et al*., 2016). iv) Analysis of isolated, specific CNS cell populations (e.g., microglia, neurons) should also be an area for further investigation. As well, analysis of a range of ages across the lifespan would enable determination of whether these age‐related changes slowly accumulate with time or if there are periods in the lifespan with more abrupt changes in genomic methylation patterns.

In summary, our results present novel evidence for sexual‐divergent DNA methylation patterns with hippocampal aging in patterns of CG and CH methylation, lifelong sex differences in CG and CH methylation and confirm the CG findings in human methylation data. The NIH recommends inclusion of animals of both sexes in studies when warranted, and these results provide a clear rationale for the need to examine both males and females and perform single‐base resolution analysis of DNA methylation patterns. Furthermore, our data highlight the complexity of the regulation and functional significance of epigenetics with aging, specifically DNA methylation, in the CNS.

## Experimental procedures

Detailed descriptions of experimental procedures, reagents, and associated references can be found in the online supporting information (Appendix S1).

### Animals

All animal experiments were performed according to protocols approved by the Penn State University Institutional Animal Care and Use Committee. Male (*N* = 8, *n* = 4 young and *n* = 4 old) and female (*N* = 8, *n* = 4 young and *n* = 4 old) C57BL6 mice ages 3 (young) and 24 (old) months were purchased from the National Institute on Aging colony at Charles River Laboratories (Wilmington, MA, USA). Mice were housed in the specific pathogen‐free Pennsylvania State University College of Medicine Hershey Center for Applied Research facility in ventilated HEPA‐filtered cages with *ad libitum* access to sterile chow (Harlan 2 918 irradiated diet, Indianapolis, IN, USA) and water. While in the facility, all animals were free of helicobacter and parvovirus. Following a 1 week acclimation period on entering the respective facility, male mice were euthanized by decapitation. Female mice were euthanized by decapitation during diestrus after estrous cycle staging.

## Funding

This work was supported by the Donald W. Reynolds Foundation, the Oklahoma Nathan Shock Center of Excellence in the Biology of Aging Targeted DNA Methylation and Mitochondrial Heteroplasmy Core (P30AG050911), the National Institute on Aging (R01AG026607, F31AG038285, T32AG052363), National Eye Institute (R01EY021716, R21EY024520, T32EY023202), Oklahoma Center for Advancement of Science and Technology (HR14‐174), and Oklahoma Center for Adult Stem Cell Research.

## Conflict of Interest

The authors have no conflicting financial interests.

## Authors' contributions

DRM, CAM, AR, and WMF designed and executed the study. DRM, NH, AU, CBG, HLP, MGD, JDW, DRS, and WMF analyzed the data. MMF provided animal support. All authors read and approved this manuscript.

## Supporting information


**Fig. S1** Definitions of sex difference and sex divergence.
**Fig. S2** Bisulfite Oligonucleotide Capture Sequencing (BOCS) method.
**Fig. S3** Bisulfite Oligonucleotide Capture Sequencing (BOCS) metrics.
**Fig. S4** Sample‐sample correlations.
**Fig. S5** Global mean methylation with age in female and male hippocampus.

**Data S1** Age‐related methylation differences at CG sites and CH sites.

**Data S2** Autosomal lifelong sex differences in CG and CH sites.

**Appendix S1**. Genomic Coordinates of dmCs.
