# Supplementary material for: Sexually divergent DNA methylation patterns with hippocampal aging
Source: Aging Cell. 2017 Sep 25;16(6):1342–52. doi: 10.1111/acel.12681 (PMC5676057; doi:10.1111/acel.12681)
Supplement: Supplementary file 1 — Fig. S1 Definitions of sex difference and sex divergence. Fig. S2 Bisulfite Oligonucleotide Capture Sequencing (BOCS) method. Fig. S3 Bisulfite Oligonucleotide Capture Sequencing (BOCS) metrics. Fig. S4 Sample‐sample correlations. Fig. S5 Global mean methylation with age in female and male hippocampus. [file ACEL-16-1342-s001.pdf]

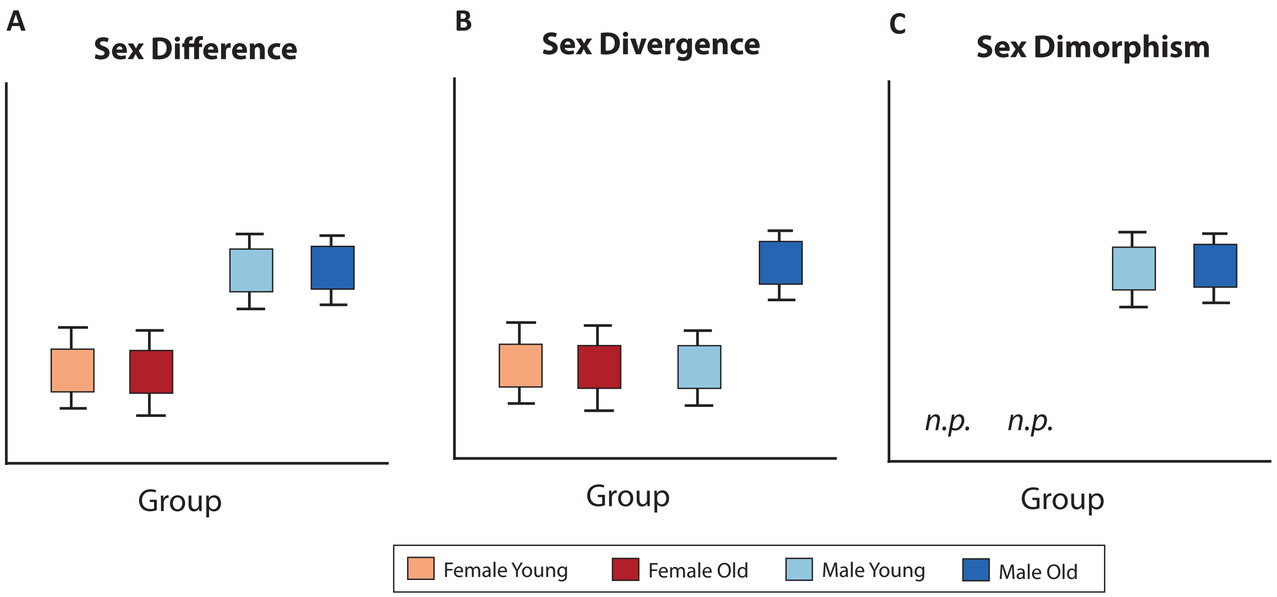

Supplmental Figure 1: Terminology definitions A) Sex differences are defined as a difference in the mean between males and females and for the purposes of this study independant of age, i.e. life-long. B) Sex divergence is a difference in response to aging that is only present in one sex, in this example an increase in males only with aging. Sex divergences can occur in either sex and can be increases or decreases. C) Sex dimorphisms are a dialectic difference between males and females where the endpoint is present in one sex, in this case males, and absent (not present, *n.p.*) in the other sex. Examples of sex dimorphisms include sex organs which are present in only one sex. Sex dimorphisms are not relavent for the present study as the cytosines examined are present in both males and females, only the level of methylation is different.

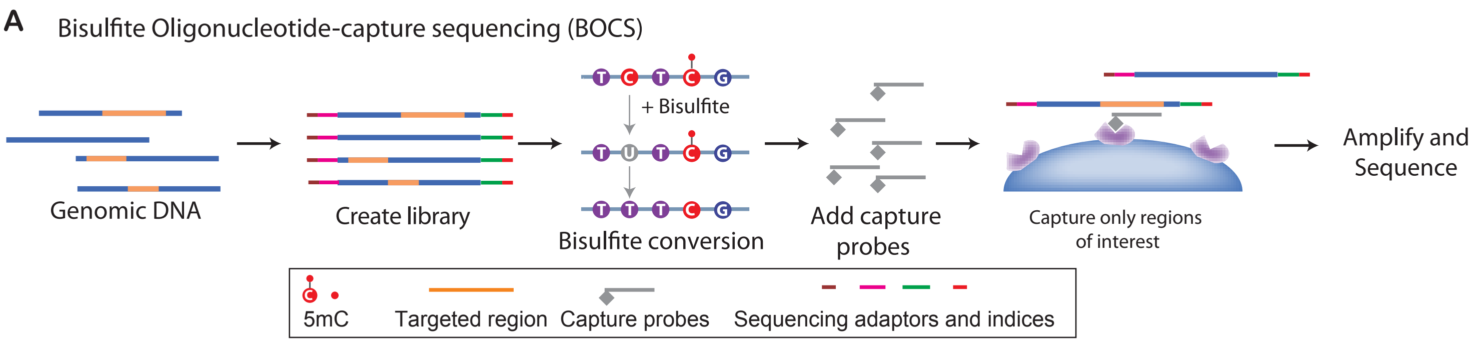

B

**Target regions**	109 Mb
Gencode/RefSeq promoters	21,267
CG islands/shores/shelves	15,952
Total CG sites	~3 million
Total CH sites	~28 million
Total # of capture probes	297,670

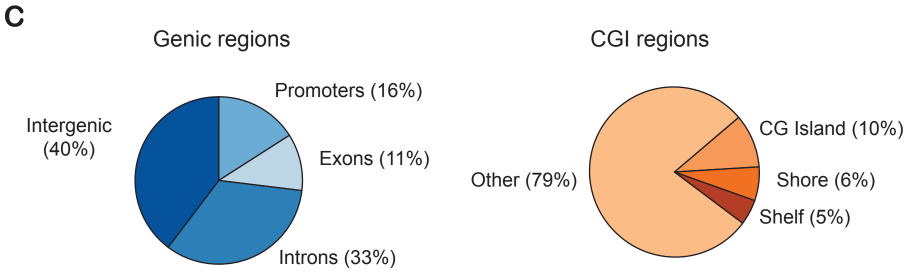

Supplemental Figure 2: Bisulfite Oligonucleotide Capture Sequencing (BOCS) method. A) The BOCS methodology utilizes genomic DNA which is made into a sequencing library followed by bisulfite conversion. After conversion the target genomic regions of interest are captured with probes against methylated and unmethylated versions of the targeted genomic regions. The target regions are then captured and amplified prior to sequencing. B) Targeted regions of the mouse genome include 109Mb containing most of the annotated promoters and CpG islands. In total nearly 3 million CG and 28 million CH sites are in the targeted regions. C) Distributions of the targeted regions according to genic and CpG island context.

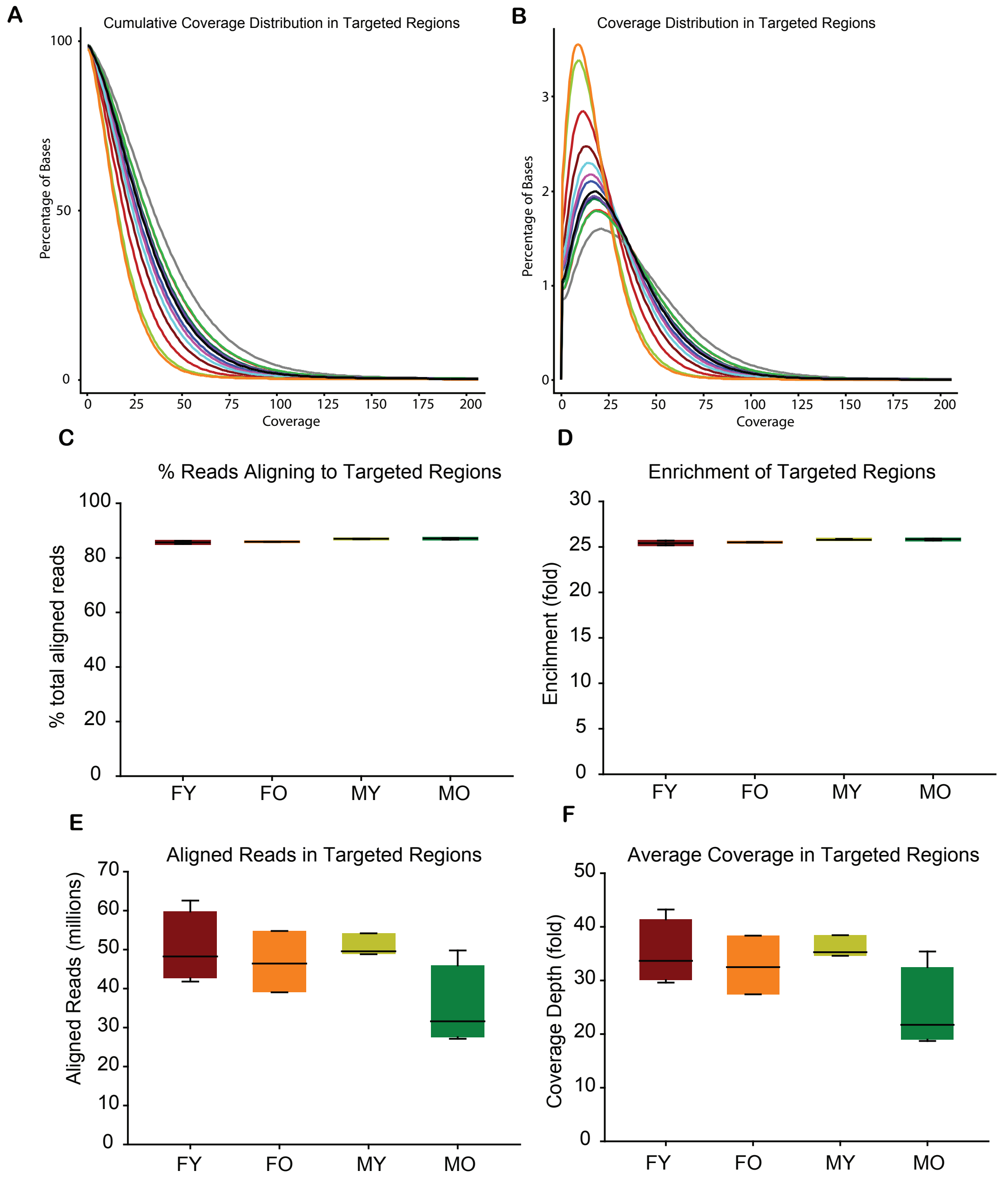

Supplemental Figure 3: Bisulfite oligonucleotide capture sequencing metrics. After aligning reads to the full mm10 genome, for the portion of the genome targeted, a cumulative coverage distribution (A) and coverage distribution (B) for the base-level coverage depth in the targeted regions was generated. Each line represents an individual sample. C) A high percentage of aligned reads corresponded to the targeted regions demonstrating specificity of oligonucleotide capture. D) Average fold enrichment, coverage in targeted regions as opposed to the rest of the genome was greater than 25 fold in all groups. Total number of aligned reads (E) and average fold base coverage (F) for the targeted regions are also presented.

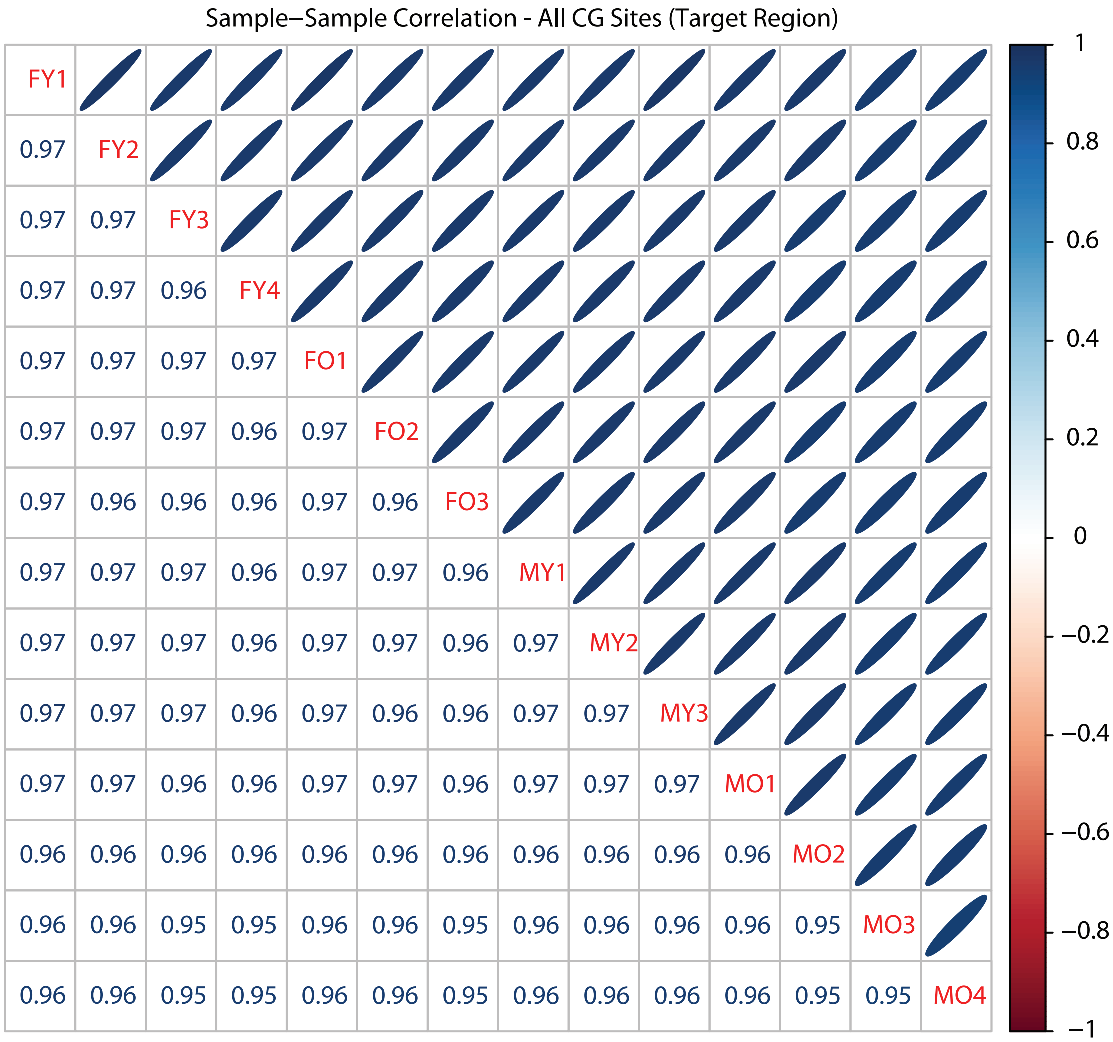

Supplemental Figure 4: Sample-sample correlations. All pair-wise sample-sample correlations are presented for the full data set. All CG sites meeting coverage requirements were used in the correlation. All samples showeda correlation of 0.95 or higher demonstrating a high degree of technical reproducibility.

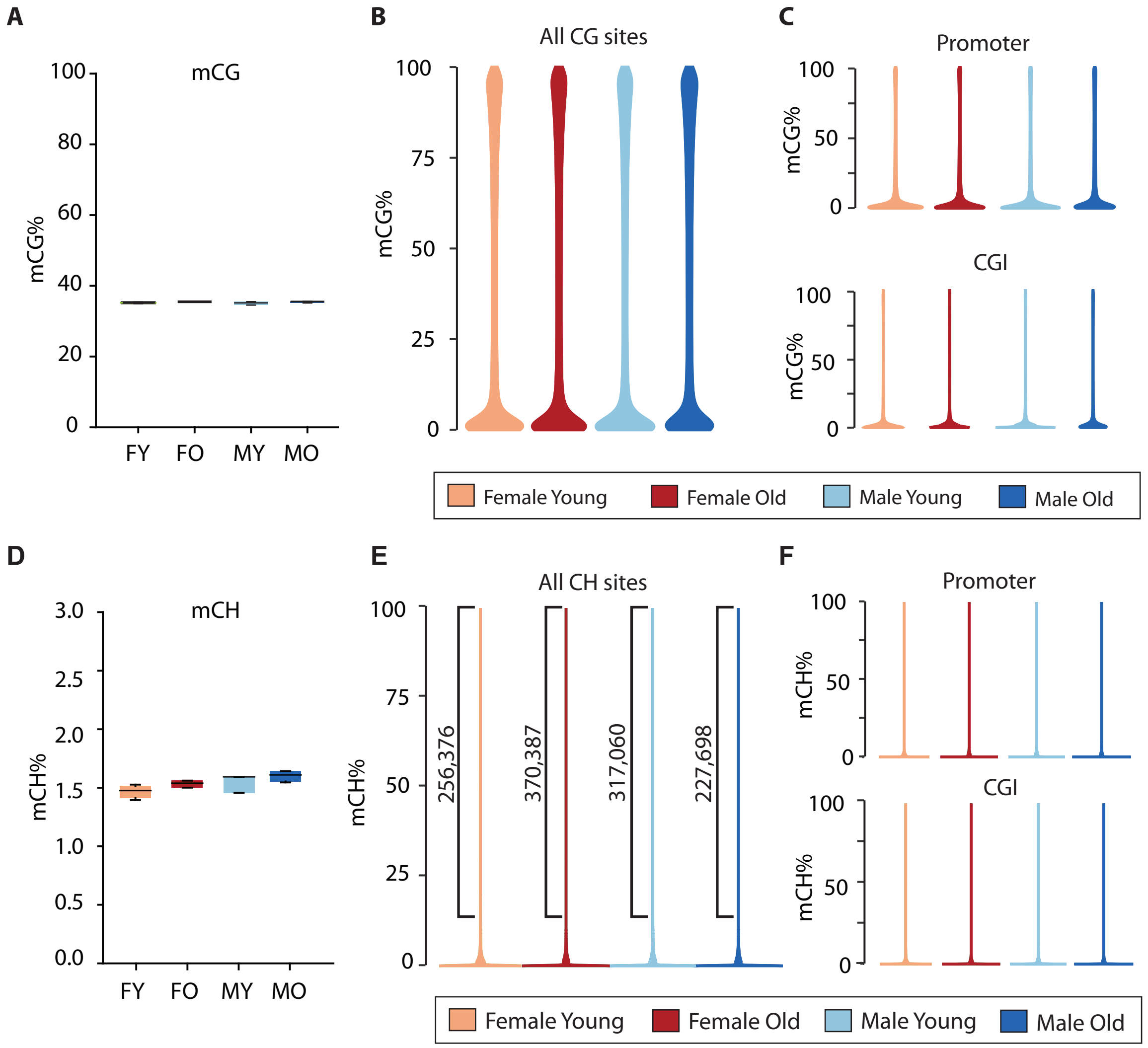

Supplemental Figure 5: Global mean methylation with age in female and male hippocampus. A) Average CG methylation in young female (FY, 35.4%), old female (FO, 35.6%), young male (MY, 35.3%), and old male (MO, 35.3%) of all single site methylation calls (mean methylation at CG sites within targeted regions) demonstrated no differences with age or sex (Two-Way ANOVA with Age and Sex as factors, Student-Newman-Keuls post-hoc (SNK), Age p=0.216, Sex p=0.323, Sex and Age p=0.730, n=3-4/group, N=14). B) Density distribution of all CG sites genome-wide, in promoters C), and in CG Islands. D) CH (H is either A, C, or T) methylation levels in FY, FO, MY, and MO were equivalent across all groups and demonstrated no differences by age or sex. E) Density distribution of CH methylation genome-wide. Brackets represent the number of CH sites from the portion of distribution in each group (FY – 256,376, FO – 370,387, MY 317,060, MO – 227,698) that have CH methylation ≥ 10%. F) CH site density distributions across young and old males and females in promoter (top) and CG Island (bottom) regions.
