# Supplementary material for: Sexually divergent DNA methylation patterns with hippocampal aging
Source: Aging Cell. 2017 Sep 25;16(6):1342–52. doi: 10.1111/acel.12681 (PMC5676057; doi:10.1111/acel.12681)
Supplement: Supplementary file 4 — Appendix S1. Genomic Coordinates of dmCs. [file ACEL-16-1342-s004.docx]

**Supplemental Methods**

*Animals*

All animal experiments were performed according to protocols approved by the Penn State University Institutional Animal Care and Use Committee. Male (N=8, n= 4 young and n=4 old) and female (N=8, n=4 young and n=4 old) C57BL6 mice ages 3 (young) and 24 (old) months were purchased from the National Institute on Aging colony at Charles River Laboratories (Wilmington, MA). Mice were housed in the speciﬁc pathogen-free Pennsylvania State University College of Medicine Hershey Center for Applied Research facility in ventilated HEPA filtered cages with *ad libitum* access to sterile chow (Harlan 2918 irradiated diet, Indianapolis, IN) and water. While in the facility all animals were free of helicobacter and parvovirus. Following a one week acclimation period on entering the respective facility, male mice were euthanized by decapitation. Female mice were euthanized by decapitation during diestrus after estrous cycle staging.

Estrous cycle staging was performed by daily vaginal lavage to control for cycling differences. Lavages were conducted as described previously (Mangold *et al.* 2017) and using well established methods (McLean *et al.* 2012) to prevent any unnecessary stress on the animals. Briefly, sterile filtered water was expelled and aspirated approximately 4-5 times into the vaginal canal until enough cells were obtained for cytological analysis. Water from the vaginal wash was then placed onto a glass slide, allowed to dry, then stained using 0.1% crystal violet. The estrous cycle consists of 3 major phases: proestrus (high estrogen), estrus (low estrogen), and diestrus (low estrogen). Proestrus is defined by having a predominance of round, nucleated epithelial cells, estrus by cornified squamous epithelial cells, and diestrus by leukocytes with few epithelial cells present (McLean *et al.* 2012).

*Bisulfite oligonucleotide-capture sequencing (BOCS)*

Genomic DNA (gDNA) was isolated from flash hippocampal frozen tissue using silica spin-columns (Zymo Duet) as described previously (Masser *et al.* 2016; Mangold *et al.* 2017). gDNA was quantified by fluorescent assay (PicoGreen, Invitrogen). 3 µg of gDNA for each sample was brought up to 50 µl volume with 1X TE and sheared by sonication (Covaris S2) to an average basepair size of 160 using the following settings; intensity of 5, duty cycle of 10%, 200 cycles per burst, 6 cycles of 60 seconds, at 4 °C. The size of sheared products was confirmed by capillary electrophoresis (DNA 1000, Agilent). gDNA libraries (SureSelect XT Methyl-Seq) were then constructed (Additional file 1: Figure S1A) following the manufacturer’s instructions (Agilent). gDNA fragments were end-repaired in 1X XT2 End-Repair Master Mix, and incubated for 30 minutes at 20 °C. End-repaired gDNA fragments were cleaned by magnetic SPRI-bead method (AMPure XP, Beckman-Coulter) and confirmed to be between 140 and 180 basepairs by capillary electrophoresis (DNA 1000). Fragments were then 3’ adenylated in 1X XT2 dA-Tailing Master Mix and incubated for 30 minutes at 37 °C. Methylated adapters were ligated on the gDNA fragments in XT2 Ligation Master Mix, incubated for 15 minutes at 20 °C, cleaned, and confirmed to be between 170 and 230 basepairs by capillary electrophoresis (DNA 1000). gDNA libraries were hybridized to the SureSelect Mouse Methyl-Seq Capture Library (Hing *et al.* 2015) (Agilent) for 24 hours at 65 °C. Capture baits were targeted for CGI units [CG Island ± 4kb (shores and shelves)], Gencode promoters, RefSeq promoters, and regulatory features including DNase I hypersensitivity sites and Ensembl regulatory features (Additional file 1: Figure S2B). Capture Library-hybridized DNA libraries were isolated and eluted using streptavidin beads. Captured DNA libraries were bisulfite converted using the EZ DNA Methylation-Gold method (Zymo Research), PCR amplified for 8 cycles, and bead purified. Libraries were indexed by PCR for 6 cycles, purified, and confirmed to be a library size of approximately 220 bp (High Sensitivity DNA Chip, Agilent). One old female and one young male library failed QC and were not included in the analysis. Libraries were quantified using standard curve qPCR (KAPA Biosystems), diluted to 4nM and pooled at equal volumes. Pooled libraries (12pM) were sequenced using the Illumina HiSeq 2500 and 2x150 Paired-End Rapid Run. All raw fastq files are publicly available from the NCBI Sequence Read Archive with the accession number PRJNA290881.

*Bioinformatics*

Prior to alignment paired-end reads were adapter trimmed and filtered in CLC Genomics Workbench 8.5.1., while end-trimming removed 3 bp and 4 bp from the 5’ and 3’ end of each paired-end read, respectively. Only reads with a Q-score >30 were used for mapping, reads which did not meet criteria after trimming were discarded. Alignment of trimmed bisulfite converted sequences was carried out using Bismark Bisulfite Mapper v0.14.4 (Krueger & Andrews 2011) against the mouse reference genome (GRCm38/mm10). Our data analysis pipeline included samtools (Li *et al.* 2009), bedtools (Quinlan & Hall 2010), and R scripts while analysis of methylation was primarily done in R v3.3.0 using the package MethylKit (Akalin *et al.* 2012).

Total mean methylation levels for CGs and CHs across the whole genome by counts were calculated averaging all per site methylation calls (CGs and CHs separately) which were determined by dividing the methylated counts over total read counts for each site. The mean methylation fraction was then multiplied by 100 (mean methylation call = (methylated counts /total read counts) X 100). Only cytosines in the BOCS targeted regions, covered by at least 10 reads, and that were present in all samples within a comparison were included for statistical analysis of age changes and sex differences, resulting in 908,118 CGs and 13,423,219 CHs analyzed for differential methylation.

Differentially methylated cytosines (DMCG and DMCH) were determined from those sites passing coverage criteria. Differentially methylated sites between groups were determined using logistic regression (Akalin *et al.* 2012). P-values were adjusted using a false-discovery rate, or FDR (Benjamini & Hochberg 1995). Only sites with a q-value < 0.05 and an absolute methylation difference of ≥ 5% were considered significant. Sex differences were limited to those sites with coordinate differential methylation at both young and old ages.

Differentially methylated sites were examined for enrichment in specific genomic elements and annotated using the R package GenomicFeatures. Refseq genes and CGI unit’s coordinates were downloaded from UCSC genome browser (GRCm38/mm10) (<https://genome.ucsc.edu/>). Sites were examined for overlap with genic regions: introns, exons and promoters (defined as 3kb upstream and 300bp downstream of the transcription start site) or CGI units [CG Island including shores (± 2kb from islands), and shelves (± 2kb from shores), or CG Island ± 4kb]. Regions to which no overlap with any Refseq annotated genes was found were determined to be present in intergenic regions. Regions with no overlap with CGI units were annotated as ‘other’. Two-tailed χ^2^ test (alpha = 0.05) was used to determine the statistically significant differences in observed versus expected genomic annotation frequencies of differential methylated sites. Data is presented as fractions related to the total distribution of the sites analyzed.

Epigenomic enrichment analysis was performed using GenomeRunner as described previously (Dozmorov *et al.* 2016). Briefly, the enrichment analysis evaluates whether sites co-localizes with genome annotation datasets in a statistically significant manner. As the NCBI37/mm9 mouse genome assembly remains the best source of genome annotation datasets, the enrichment analysis was performed using mm9 genomic coordinates of ENCODE data from the UCSC genome browser database (Karolchik *et al.* 2014), accessed 07-22-2015 and a liftover from mm10 to mm9 for the DMCGs and DMCHs. The two-tailed χ^2^ test was used to calculate enrichment/depletion p-values, corrected for multiple testing using False Discovery Rate (FDR) approach, and odds ratios (limiting to only odds ratios <0.9 and >1.1).

Human brain methylation data were obtained from NCBI Gene Expression Omnibus (GEO), specifically control (not known to be diseased) samples from series GSE89703 (Viana *et al.* 2017) and GSE63347 (Horvath *et al.* 2015) for hippocampal data (22 samples, ages 25-95 years) and GSE41826 (Guintivano *et al.* 2013) for frontal cortex data (145 samples, ages 13-79). All data were generated from Illumina 450k Methylation Arrays and deposited onto GEO as beta values. Beta values and sample annotations were collected from GEO and no normalization or corrections were applied; rows with any missing values (NAs), cross-reactive probes (Chen *et al.* 2012), and all sex chromosome sites were removed from analyses leaving 413085 probes for analysis in hippocampal samples and 469680 probes for analysis in frontal cortex. A set of general linear models (Ritchie *et al.* 2015) were used to observe the effects of age on methylation in hippocampus and frontal cortex (Figure 5 Panel A) and to test the main effects of age on methylation and sex as well as a model of the sex by age interaction for the frontal cortex (limiting to 69 male and 70) females samples ranging from 13-60 years of age, Figure 5 Panel B-E). Multiple testing correction was done for false discovery rate and a corrected p value of less than 0.05 was considered significant. All statistics were done using R statistics platform (Team 2015) and plotted using ggplot2 (Wickham 2009) and associated themes (Arnold 2017).

Supplemental References:

Akalin A, Kormaksson M, Li S, Garrett-Bakelman FE, Figueroa ME, Melnick A, Mason CE (2012). methylKit: a comprehensive R package for the analysis of genome-wide DNA methylation profiles. *Genome Biol*. **13**, R87.

Arnold JB (2017). Ggthemes: Extra Themes, Scales and Geoms for 'ggplot2'ed^eds).

Benjamini Y, Hochberg Y (1995). Controlling the False Discovery Rate - a Practical and Powerful Approach to Multiple Testing. *J Roy Stat Soc B Met*. **57**, 289-300.

Chen YA, Choufani S, Grafodatskaya D, Butcher DT, Ferreira JC, Weksberg R (2012). Cross-reactive DNA microarray probes lead to false discovery of autosomal sex-associated DNA methylation. *Am J Hum Genet*. **91**, 762-764.

Dozmorov MG, Cara LR, Giles CB, Wren JD (2016). GenomeRunner web server: regulatory similarity and differences define the functional impact of SNP sets. *Bioinformatics*. **32**, 2256-2263.

Guintivano J, Aryee MJ, Kaminsky ZA (2013). A cell epigenotype specific model for the correction of brain cellular heterogeneity bias and its application to age, brain region and major depression. *Epigenetics*. **8**, 290-302.

Hing B, Ramos E, Braun P, McKane M, Jancic D, Tamashiro KL, Lee RS, Michaelson JJ, Druley TE, Potash JB (2015). Adaptation of the targeted capture Methyl-Seq platform for the mouse genome identifies novel tissue-specific DNA methylation patterns of genes involved in neurodevelopment. *Epigenetics*. **10**, 581-596.

Horvath S, Garagnani P, Bacalini MG, Pirazzini C, Salvioli S, Gentilini D, Di Blasio AM, Giuliani C, Tung S, Vinters HV, Franceschi C (2015). Accelerated epigenetic aging in Down syndrome. *Aging Cell*. **14**, 491-495.

Karolchik D, Barber GP, Casper J, Clawson H, Cline MS, Diekhans M, Dreszer TR, Fujita PA, Guruvadoo L, Haeussler M, Harte RA, Heitner S, Hinrichs AS, Learned K, Lee BT, Li CH, Raney BJ, Rhead B, Rosenbloom KR, Sloan CA, Speir ML, Zweig AS, Haussler D, Kuhn RM, Kent WJ (2014). The UCSC Genome Browser database: 2014 update. *Nucleic acids research*. **42**, D764-770.

Krueger F, Andrews SR (2011). Bismark: a flexible aligner and methylation caller for Bisulfite-Seq applications. *Bioinformatics*. **27**, 1571-1572.

Li H, Handsaker B, Wysoker A, Fennell T, Ruan J, Homer N, Marth G, Abecasis G, Durbin R, Genome Project Data Processing S (2009). The Sequence Alignment/Map format and SAMtools. *Bioinformatics*. **25**, 2078-2079.

Mangold CA, Masser DR, Stanford DR, Bixler GV, Pisupati A, Giles CB, Wren JD, Ford MM, Sonntag WE, Freeman WM (2017). CNS-wide Sexually Dimorphic Induction of the Major Histocompatibility Complex 1 Pathway With Aging. *J Gerontol A Biol Sci Med Sci*. **72**, 16-29.

Masser DR, Stanford DR, Hadad N, Giles CB, Wren JD, Sonntag WE, Richardson A, Freeman WM (2016). Bisulfite oligonucleotide-capture sequencing for targeted base- and strand-specific absolute 5-methylcytosine quantitation. *Age (Dordr)*. **38**, 49.

McLean AC, Valenzuela N, Fai S, Bennett SA (2012). Performing vaginal lavage, crystal violet staining, and vaginal cytological evaluation for mouse estrous cycle staging identification. *Journal of visualized experiments : JoVE*, e4389.

Quinlan AR, Hall IM (2010). BEDTools: a flexible suite of utilities for comparing genomic features. *Bioinformatics*. **26**, 841-842.

Ritchie ME, Phipson B, Wu D, Hu Y, Law CW, Shi W, Smyth GK (2015). limma powers differential expression analyses for RNA-sequencing and microarray studies. *Nucleic acids research*. **43**, e47.

Team RC (2015). R: A language and envrionment for statistical computinged^eds). Vienna, Austria: R foundation for Statistical Computing.

Viana J, Hannon E, Dempster E, Pidsley R, Macdonald R, Knox O, Spiers H, Troakes C, Al-Saraj S, Turecki G, Schalkwyk LC, Mill J (2017). Schizophrenia-associated methylomic variation: molecular signatures of disease and polygenic risk burden across multiple brain regions. *Hum Mol Genet*. **26**, 210-225.

Wickham H (2009). *ggplot2: Elegant Graphics for Data Analysis*. New York: Springer-Verlag.
